# Tricetin Protects Rat Chondrocytes against IL-1*β*-Induced Inflammation and Apoptosis

**DOI:** 10.1155/2019/4695381

**Published:** 2019-04-28

**Authors:** Fang-Fang Sun, Peng-Fei Hu, Yan Xiong, Jia-Peng Bao, Jing Qian, Li-Dong Wu

**Affiliations:** ^1^Key Laboratory of Cancer Prevention and Intervention, China National Ministry of Education, The Second Affiliated Hospital, Cancer Institute, School of Medicine, Zhejiang University, Hangzhou, Zhejiang 310009, China; ^2^Department of Orthopedic Surgery, The Second Affiliated Hospital, School of Medicine, Zhejiang University, Hangzhou, Zhejiang 310009, China; ^3^Orthopedics Research Institute of Zhejiang University, Hangzhou, Zhejiang 310009, China; ^4^Pharmaceutical Informatics Institute, College of Pharmaceutical Sciences, Zhejiang University, Hangzhou, Zhejiang 310058, China

## Abstract

Tricetin is a well-studied flavonoid with a wide range of pharmacological activities in cancer and inflammation. However, the ability of tricetin to ameliorate the inflammation that occurs in osteoarthritis (OA) has not been determined. This study explored the effects of tricetin on interleukin- (IL-) 1*β*-induced rat chondrocytes. Chondrocytes harvested from rat cartilage were incubated in vitro with tricetin in the presence of IL-1*β*. The expression of matrix metalloproteinase- (MMP-) 1, MMP-3, MMP-13, nitric oxide (NO), prostaglandin E2 (PGE2), Bax, and Bcl-2 was evaluated by real-time-PCR, ELISA, Griess reaction, and western blotting. Caspase-3 activity in chondrocytes was determined using a caspase-3 activity assay and MAPK pathway activity by western blotting. Tricetin decreased the expression of MMP-1, MMP-3, and MMP-13 at both the gene and protein level in IL-1*β*-induced rat chondrocytes. It also inhibited IL-1*β*-induced NO and PGE2 production, by modulating inducible NO synthase and cyclooxygenase 2 gene expression. An antiapoptotic role of tricetin involving the Bax/Bcl-2/caspase-3 pathway was also determined. The chondroprotective effect of tricetin was shown to be partly related to the suppression of the MAPK signaling pathway. The results of this study demonstrate the chondroprotective role of tricetin, based on its anticatabolic, anti-inflammatory, and antiapoptotic effects in chondrocytes. The therapeutic potential of tricetin in OA patients should be explored in future studies.

## 1. Introduction

As the average age of populations throughout the world is steadily rising, osteoarthritis (OA) has become an increasingly large economic burden in many countries [1]. OA is a chronic, degenerative joint disease that causes substantial pain and functional disability. Inflammation of the joint tissue, degradation of the extracellular matrix (ECM), oxidative stress, and chondrocyte apoptosis all contribute substantially to the pathogenesis of OA [2, 3]. In OA, the activities of catabolic factors, such as matrix metalloproteinases (MMPs) and a disintegrin and metalloproteinase with thrombospondin motifs (ADAMTS), as well as of inflammatory cytokines, such as interleukin- (IL-) 1*β*, IL-6, and tumor necrosis factor- (TNF-) *α*, result in cartilage erosion and bone damage [4, 5]. Chondrocytes are the only resident cells in the articular cartilage, and they contribute to maintaining cartilage metabolism. Although the primary cause of OA is still not fully understood, the apoptosis of chondrocytes has been shown to play an essential role in disease initiation. Specifically, in the previous study, the percentage of apoptotic chondrocytes was higher in cartilage samples obtained from OA patients than in normal cartilage (18–21% vs. 2–5%) [6]. Articular cartilage is avascular, with no cell turnover, and the degree of articular erosion is positively associated with chondrocyte apoptosis [7]. Given the complexity of the pathophysiology of OA, very few effective disease-modifying drugs are currently available for clinical use, and the search for new drugs able to prevent OA progression continues.

In recent years, the therapeutic potential of natural plant products has received increasing attention. Flavonoids, a large class of polyphenolic plant compounds, are present in fruit, vegetables, chocolate, and beverages and can be easily extracted from these products [8]. Moreover, several studies have reported the anti-inflammatory, antithrombogenic, antidiabetic, anticancer, and neuroprotective activities of flavonoids via diverse mechanisms of action [9–13]. There is also evidence of a therapeutic effect of flavonoids in OA. Chen et al. reported that licochalcone A, a classical flavonoid separated from licorice, inhibits the expression of MMP-1, MMP-13, ADAMTS4, and ADAMTS5 in IL-1*β*-induced chondrocytes, by regulating the NF-*κ*B and wnt/*β*-catenin signaling pathways [14]. Chondroprotective effects were also reported for the flavonoid silibinin [15], in a study showing that it significantly decreased the IL-1*β*-induced expression of proinflammatory mediators and MMPs in human OA chondrocytes. The present study investigated the flavonoid tricetin (5,7,3′,4′,5′-pentahydroxyflavone), obtained from Myrtaceae pollen and eucalyptus honey [16]. The many biological actions of tricetin include anticancer and anti-inflammatory activities [17, 18]. However, whether tricetin is effective in treating OA has not been investigated. Thus, using rat chondrocytes, we examined the pharmaceutical role of tricetin in the treatment of OA by determining the expression of inflammatory and apoptotic mediators in chondrocytes pretreated with tricetin. The mechanisms underlying the observed actions of tricetin in this OA model were also explored.

## 2. Materials and Methods

### 2.1. Reagents

Tricetin (purity ≥ 99%) was obtained from Extrasynthese, Genay, France. Recombinant rat IL-1*β*, MTT, and type II collagenase were purchased from Sigma-Aldrich, Merck KGaA, Darmstadt, Germany. Dulbecco's Modified Eagle's Medium (DMEM), penicillin, streptomycin, fetal calf serum (FBS), 0.25% pancreatic enzyme, TRIzol reagent, and cDNA synthesis kit were all purchased from Thermo Fisher Scientific Inc. PGE2 ELISA kit was purchased from R&D Systems, Minneapolis, MN, USA. Caspase-3 cellular activity assay kit, cell lysis buffer, and antibodies against ERK1/2, p-ERK1/2, p38, p-p38, JNK, and p-JNK were purchased from Cell Signaling Technology, Danvers, MA, USA. Antibodies against MMP-3, MMP-13, Bcl-2, and Bax were purchased from Abcam, Cambridge, USA. Antibody against MMP-1 was purchased from Proteintech Group Inc., Rosemont, USA. Griess reagent was purchased from Beyotime, Shanghai, China. SYBR Premix Ex Taq II was purchased from Takara Biotechnology, Dalian, China.

### 2.2. Cell Culture and Treatment

The study was approved by the Ethics Committee of the Second Affiliated Hospital, Zhejiang University School of Medicine, Hangzhou, China (2019-01). Cartilage pieces obtained from the hip and knee joints of 6-week-old Sprague-Dawley (SD) rats (24 rats; sex ratio: 1 : 1) were digested with 0.25% pancreatic enzyme for 30 min, followed by 0.2% collagenase II for 2 h. The chondrocytes collected from the digest were cultured as a monolayer in DMEM supplemented with 10% fetal calf serum, penicillin (100 U/ml) and streptomycin (100 g/ml) at 37°C in an atmosphere of 5% CO_2_.

### 2.3. Cell Viability Assay

**Figure 1:**
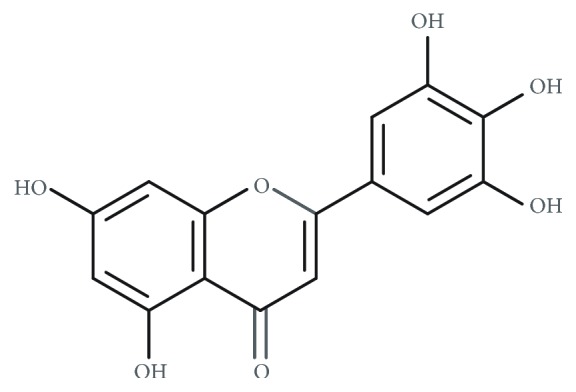
Chemical structure of tricetin.

The cytotoxicity of tricetin in the chondrocyte cultures was determined in an MTT assay, conducted to select a nontoxic concentration range for use in the subsequent experiments. The assay was performed according to the manufacturer's instructions. Chondrocytes (5 × 10^3^ cells/well) seeded in 96-well plates were incubated with various concentrations of tricetin (Figure 1) for 24 h at 37°C and then with 20 *μ*l MTT (5 mg/ml) for 4 h at 37°C. The resulting MTT formazan crystals were solubilized with an equal volume of dimethyl sulfoxide (DMSO). The absorbance of the contents of each well was measured at 570 nm using a microplate reader. Concentrations that did not result in a significantly toxic effect were selected for further use in the experiments.

### 2.4. Quantitative Real-Time Polymerase Chain Reaction (qRT-PCR)

The rats were divided into four groups: control, IL-1*β*, IL-1*β*+low-dose (10 *μ*m) tricetin, and IL-1*β*+high-dose (20 *μ*M) tricetin. Chondrocytes (5 × 10^5^ cells/well) were seeded in 6-well plates and stimulated or not with tricetin for 3 h. IL-1*β* (10 ng/ml) was then added to the wells of all groups except for the control. After 24 h of incubation. The cultures were assayed by real-time PCR (RT-PCR) for the expression of MMP-1, MMP-3, MMP-13, inducible NO synthase (iNOS), cyclooxygenase 2 (COX-2), Bcl-2, and Bax. Total RNA was extracted from all four groups using the TRIzol reagent according to the manufacturer's instructions, and 1 *μ*g RNA was reverse transcribed using the cDNA synthesis kit. RT-PCR was performed using the SYBR Premix Ex Taq II system as follows: 45 cycles of 95°C for 15 s and 60°C for 30 s. The sequences of the primers used in the reaction are listed in Table 1. Relative gene expression was calculated using the 2^−*∆∆*Ct^ method. GAPDH served as the internal control gene.

### 2.5. ELISA and the Griess Reaction

Cell culture supernatants were assayed for prostaglandin E2 (PGE2) expression using an ELISA kit according to the manufacturer's instructions. All assays were performed in duplicate. Nitric oxide (NO) production was measured by quantifying the accumulation of nitrite, a stable end product, according to the Griess reaction, and performed as follows: 100 *μ*l of the culture medium was mixed with an equal volume of the Griess reagent (1% sulfanilamide/0.1% naphthylethyllene dihydrochloride/2.5% phosphoric acid). After 15 min at room temperature, the absorbance of the reaction at 540 nm was determined. All assays were performed in duplicate.

### 2.6. Caspase-3 Activity

Chondrocytes (5 × 10^4^ cells/well) were seeded in 96-well plates and divided into four groups with the similar treatment. A caspase-3 cellular activity assay kit was used to determine caspase-3 activity in the four groups. The reaction is based on the cleavage of the fluorogenic substrate (N-acetyl-Asp-Glu-Val-Asp-7-amido-4-methylcoumarin: Ac-DEVDAMC) between the DEVD and AMC moieties by activated caspase-3. Highly fluorescent AMC is then detected at an excitation wavelength of 380 nm using a fluorescence reader. In this study, chondrocytes pretreated in the growth medium supplemented or not with low- and high-dose tricetin for 3 h were incubated or not with rat recombinant IL-1*β* (10 ng/ml) for 24 h. According to the manufacturer's protocol, the chondrocytes were collected and lysed in cell lysis buffer in the presence or absence of 5 *μ*l DEVD-pNA for 1 h at 37°C. Caspase-3 activity was measured at 405 nm on a microplate reader. The experiment was performed in triplicate.

### 2.7. Western Blotting

Chondrocytes (2 × 10^6^ cells/well) were seeded in 6-well plates and divided into four groups. In brief, chondrocytes were harvested and lysed with cell lysis buffer. The BCA protein assay kit was used to determine protein concentrations. For each sample, 20 *μ*g of protein was loaded and resolved by SDS-PAGE on a 10% polyacrylamide gel. The proteins were transferred onto a nitrocellulose membrane and incubated overnight at 4°C with primary antibodies against MMP-1, MMP-3, MMP-13, Bcl-2, Bax, ERK1/2, p-ERK1/2, p38, p-p38, JNK, and p-JNK. The membranes were then washed with TBS-T and incubated with secondary antibodies for 1 h at room temperature. Immunoreactive bands were detected using an enhanced chemiluminescence assay (Fude Biological Technology, Hangzhou, China) and exposed on Kodak X-Omat film (Kodak, Rochester, NY, USA).

### 2.8. Statistical Analysis

The data are reported as mean ± standard deviation (SD). All experiments were performed at least three times. A one-way analysis of variance and post hoc Tukey's test were performed using SPSS (ver. 19.0; IBM Corp., Armonk, NY, USA) and GraphPad Prism 7 (GraphPad Software Inc., La Jolla, CA, USA) software. For all analyses, a *p* value < 0.05 was considered to indicate statistical significance.

## 3. Results

### 3.1. Effects of Tricetin on Cell Viability

**Figure 2:**
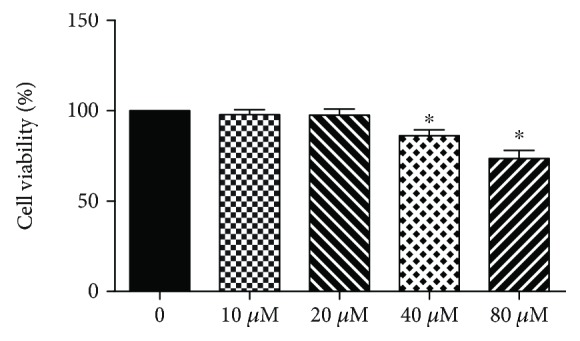
Effects of tricetin on cell viability as evaluated by MTT assay. Chondrocytes were treated with 10–80 *μ*M tricetin for 24 h. Cells incubated in the medium without tricetin were used as the control, and their viability was set at 100%. The values are expressed as mean ± SD from triplicate samples in three independent experiments. ^∗^*p* < 0.05 vs. the control group.

As shown in Figure 2, tricetin at a concentration of 0–20 *μ*M exhibited no significant cytotoxicity to the cultured chondrocytes. Higher concentrations of tricetin (40 and 80 *μ*M) inhibited chondrocyte proliferation compared to nontreated cells. Therefore, 10 and 20 *μ*M concentrations of tricetin were viewed as safe concentrations and used in the following experiments.

### 3.2. Effects of Tricetin on MMP-1, MMP-3, and MMP-13 Expression in Chondrocytes

**Figure 3:**
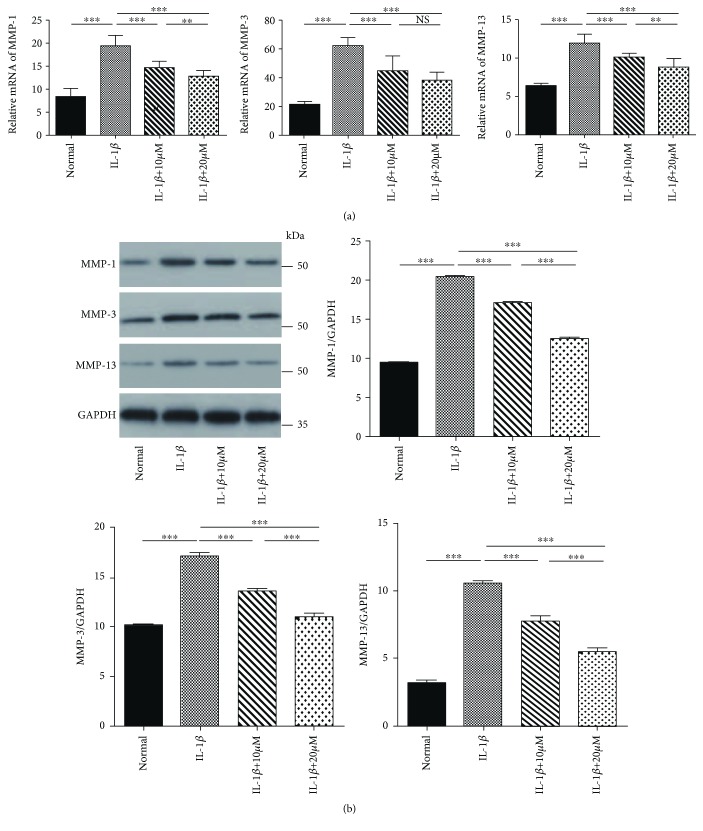
Effects of tricetin on the expression of matrix metalloproteinase- (MMP-) 1, MMP-3, and MMP-13, as determined by real-time PCR (RT-PCR) (a) and western blot (b). Chondrocytes pretreated with 10 or 20 *μ*M tricetin for 3 h were cultured with interleukin- (IL-) 1*β* (10 ng/ml) for 24 h. Each column represents the mean ± SD from triplicate samples in three independent experiments. IL-1*β* (10 ng/ml) alone significantly increased MMP-1, MMP-3, and MMP-13 expression compared to the untreated group (*p* < 0.05). Tricetin (10 or 20 *μ*M) decreased IL-1*β*-induced MMP production. ^∗∗∗^*p* < 0.001 and ^∗∗^*p* < 0.01; NS: not significant.

The effect of tricetin on MMP-1, MMP-3, and MMP-13 mRNA expression was assessed using RT-PCR (Figure 3(a)). The results showed that IL-1*β* (10 ng/ml) significantly increased the expression of all three enzymes compared to the untreated control group, in accordance with the previous report [19]. The anticatabolic effect of tricetin at different concentrations was evidenced by its reduction of IL-1*β*-induced MMP expression. On the western blot (Figure 3(b)), the changes in MMP-1, MMP-3, and MMP-13 protein expression were similar among the four groups, consistent with the quantitative RT-PCR results.

### 3.3. Effects of Tricetin on NO and PGE2 Production

**Figure 4:**
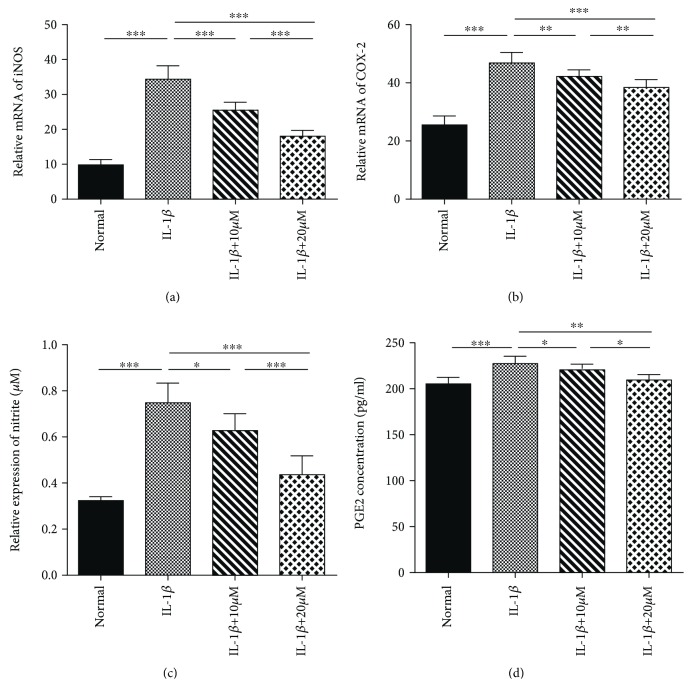
Effects of tricetin on nitric oxide (NO) and prostaglandin E2 (PGE2) production in IL-1*β*-induced chondrocytes, as determined by RT-PCR (a and b), the Griess reaction (c), and ELISA (d). Each column represents the mean ± SD from triplicate samples in three independent experiments. Tricetin (10 and 20 *μ*M) significantly inhibited both IL-1*β*-induced inducible NO synthase (iNOS) gene expression and NO production, as well as cyclooygenase 2 (COX-2) and PGE2 expression. ^∗∗∗^*p* < 0.001, ^∗∗^*p* < 0.01, and ^∗^*p* < 0.05.

The anti-inflammatory effects of tricetin were also evaluated, by measuring NO and PGE2 expression in the rat chondrocytes. According to the results of the Griess reaction and RT-PCR (Figure 4), stimulation with IL-1*β* (10 ng/ml) for 24 h led to a significant increase in both iNOS gene expression and NO production compared to the untreated group. Tricetin, at a concentration of either 10 or 20 *μ*M, decreased the IL-1*β*-induced expression of the iNOS gene and the production of NO, as well as COX-2 gene expression and PGE2 production in chondrocytes.

### 3.4. Tricetin Inhibits IL-1*β*-Induced Apoptosis by Suppressing Caspase-3 Activity

**Figure 5:**
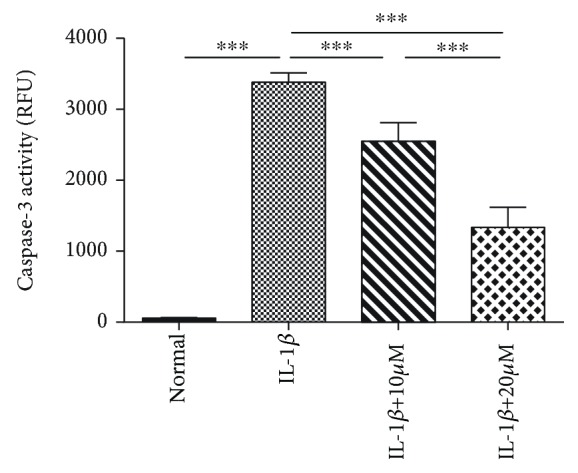
Effects of tricetin on caspase-3 activity. In the absence of tricetin, caspase-3 activity is significantly increased in IL-1*β*-stimulated chondrocytes (*p* < 0.05). Tricetin (10 and 20 *μ*M) downregulates IL-1*β*-induced caspase-3 activity. Each column represents the mean ± SD from triplicate samples in three independent experiments. ^∗∗∗^*p* < 0.001.

Caspase-3 activity increased significantly following the addition of IL-1*β*, whereas in chondrocytes treated with tricetin a marked and dose-dependent decrease was observed (Figure 5).

### 3.5. Tricetin Suppresses the Apoptotic Pathway Mediated by Bcl-2 and Bax

**Figure 6:**
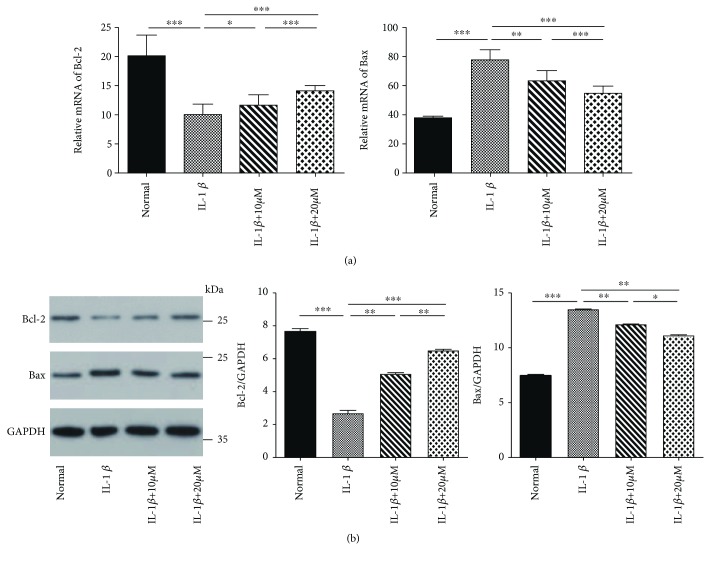
Effects of tricetin on Bcl-2 and Bax gene and protein expression, as determined by RT-PCR (a) and western blot (b). After the stimulation of rat chondrocytes with IL-1*β* alone, Bax gene and protein levels increased significantly, while Bcl-2 expression decreased. Tricetin significantly decreased Bax production and increased Bcl-2 expression in IL-1*β*-induced rat chondrocytes. Each column represents the mean ± SD from triplicate samples in three independent experiments. ^∗∗∗^*p* < 0.001, ^∗∗^*p* < 0.01, and ^∗^*p* < 0.05.

Both qRT-PCR (Figure 6(a)) and western blot (Figure 6(b)) analyses showed that IL-1*β* stimulation alone significantly increased the level of the proapoptotic factor Bax, whereas the expression of the antiapoptotic factor Bcl-2 was decreased. However, pretreatment of the chondrocytes with tricetin increased the transcript and protein levels of Bcl-2 and decreased those of Bax (*p* < 0.05).

### 3.6. Effects of Tricetin on MAPK Activation in Chondrocytes

**Figure 7:**
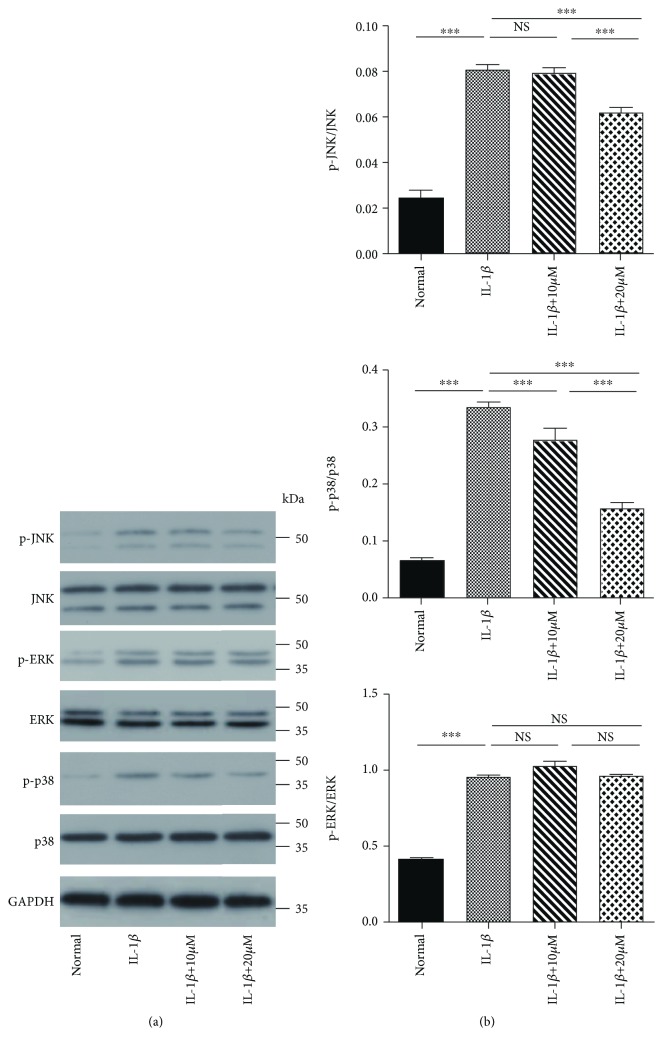
Effects of tricetin on MAPK activation in IL-1*β*-induced chondrocytes. Tricetin decreased p-JNK and p-p38 protein levels, but not the level of p-ERK in IL-1*β*-induced chondrocytes. GAPDH was used as the loading control in western blotting. Each column represents the mean ± SD from triplicate samples in three independent experiments. ^∗∗∗^*p* < 0.001, NS: not significant, and ^∗^*p* < 0.05 vs. the IL-1*β* group. ^#^*p* < 0.05 vs. the control group.

To determine the potential mechanism underlying the effect of tricetin in IL-1*β*-stimulated chondrocytes, alterations in the MAPK pathway were assessed. Compared to the control group, the levels of p-JNK, p-ERK, and p-p38 were markedly increased in the group treated with IL-1*β* alone (Figure 7). Treatment with tricetin (10 and 20 *μ*M) significantly attenuated the IL-1*β*-induced phosphorylation of p-JNK and p-p38, but not that of p-ERK. In addition, the total basal levels of JNK, p38, and ERK were unaffected by IL-1*β* and tricetin.

## 4. Discussion

Tricetin shows a wide range of pharmacological activities in several disease states, especially cancer and inflammation. Its anti-inflammatory activity in lipopolysaccharide- (LPS-) exposed kidney mesangial cells is mediated by its ability to decrease LPS-induced NO production [18]. Chang et al. found that tricetin inhibits MMP-9 expression in human osteosarcoma cells via the MAPK signaling pathway [17]. The inhibition of MMP expression has also been implicated in the antimetastatic properties of tricetin in human glioblastoma multiforme and oral cancer cells [20, 21]. These studies clearly demonstrated the strong correlation between tricetin and MMP expression. Since an increase in MMP levels is regarded as a risk factor for the erosion of the articular cartilage, in this study, we investigated the underlying effect of tricetin in chondrocytes. To the best of our knowledge, this study is the first to show that tricetin prevents IL-1*β*-induced catabolism and inflammation in chondrocytes. Moreover, an antiapoptotic role for tricetin, by regulating the Bax/Bcl-2 pathway, was also determined. This chondroprotective effect of tricetin was then linked to the MAPK signaling pathway.

In the pathological process of OA, ECM degradation, mediated by MMPs, ADAMTS, and cathepsins [22–24], plays a fundamental role. MMPs are a calcium-dependent family of catabolic enzymes responsible for cartilage degradation; they can be divided into three groups: collagenases, gelatinases, and stromelysin. Type II collagen, which together with aggrecan is the main component of the cartilage matrix, is cleaved predominantly by MMP-13 and MMP-3 and aggrecan by ADAMTS [25]. These catabolic mediators are therefore potential therapeutic targets. In MMP-13 knockout mice, reductions in tibial cartilage damage and aggrecan loss compared to wild-type mice were observed [26]. Based on these findings, we explored the anticatabolic effect of tricetin by measuring the levels of MMP-1, MMP-3, and MMP-13 in chondrocytes pretreated with IL-1*β* to simulate the inflammatory environment. IL-1*β* was a classical proinflammatory cytokine and widely used to simulate the production of MMPs and other inflammatory mediators in chondrocytes [27]. According to the previous study, IL-1*β* at a concentration of 10 ng/ml has been well accepted to mimic pathological events of OA [28]. As expected, IL-1*β* (10 ng/ml) significantly increased the expression of catabolic, inflammatory, and apoptotic factors in the treated cells, consistent with the results of the previous study [29]. However, in IL-1*β*-induced and tricetin-treated chondrocytes, MMPs were downregulated, as was the expression of the genes encoding iNOS and COX-2. Thus, the chondroprotective effect of tricetin seems to be due to its regulation of the expression of catabolic and inflammatory factors. The recent studies have also demonstrated that many members of the flavanoid family, such as anthocyanins, wogonin, and icariin, decrease catabolic marker genes and reduce extracellular matrix breakdown [30–32]. These data suggested that flavonoid administration could ameliorate the progress of OA and may be a potential therapeutic strategy for OA treatment. The dysregulation of apoptosis contributes to the development of OA. The previous study has reported that chondrocyte apoptosis and a decrease in chondrocyte survival signals were associated with the catabolic process of OA [33]. The caspase family is regarded as the key regulator of apoptosis. Caspase-3 is a key enzyme in the activation of the mitochondrial-dependent apoptosis pathway [34], and its expression is significantly increased in OA cartilage in the knee and hip compared to control tissues [35]. The Bcl family is divided into proapoptotic (Bax and the BH3-only families) and antiapoptotic (Bcl-2, Bcl-xL, Bcl-w, A1, and Mcl1) mediators. Decreased Bcl-2 levels were demonstrated in damage OA articular cartilage compared to nonlesional areas of the same tissue [36]. Similarly, chondrocytes incubated with the proapoptotic factors TNF-*α*, IL-1*β*, and interferon-*γ* had a higher Bax/Bcl-2 ratio [37]. Chondrocyte stimulation with IL-1*β* was shown to activate caspase-3 and upregulate Bax [38]. In this study, apoptosis and the antiapoptotic effect of tricetin were investigated in chondrocytes by measuring Bcl-2 and Bax levels. Pretreatment with tricetin was shown to directly increase the transcript and protein levels of Bcl-2 and decrease those of Bax. Taken together, our findings provide evidence that tricetin regulates the Bax/Bcl-2/caspase-3 pathway and thereby inhibits the apoptosis of IL-1*β*-stimulated chondrocytes.

The MAPK cascade involves a highly conserved family of serine/threonine protein kinases. The MAPK signaling pathway, which includes ERK1/2, Jnk, and p38, plays a central role in chondrocyte proliferation, apoptosis, and survival [39, 40]. In our in vitro study, we demonstrated that tricetin inhibits the phosphorylation of ERK and p38, consistent with its ability to reduce Akt and p38 phosphorylation in human osteosarcoma cells [17]. Chung et al. reported that tricetin decreases MMP activity by downregulating p38 and JNK [20]. These results suggest that tricetin acts as a ligand that binds to an upstream factor in the MAPK signaling pathway. However, the direct target of tricetin in chondrocytes remains to be identified. A limitation of our study is that our current research has not carried out any experiment of MAPK pathway silencing to prove that MAPK is fully involved in IL-1*β*-induced inflammation and apoptosis with tricetin treatment. It needs a further study to certify that the MAPK signaling pathway is the main mechanism in which tricetin protects rat chondrocytes against IL-1*β*-induced inflammation and apoptosis.

## 5. Conclusion

Our study showed that tricetin decreases the expression of MMP-1, MMP-3, and MMP-13, at both the gene and protein level, in IL-1*β*-induced rat chondrocytes. Tricetin also inhibited IL-1*β*-induced NO and PGE2 production by modulating iNOS and COX-2 gene expression, as well as apoptosis by regulating the Bax/Bcl-2/caspase-3 pathway. The chondroprotective effect of tricetin might be attributed to its suppression of the MAPK signaling pathway. Thus, tricetin should be explored as the basis for a new approach to the treatment of OA.

## Figures and Tables

**Table 1:** Real-time PCR primers and conditions.

Gene	GenBank accession	Primer sequences (5′ to 3′)	Size (bp)	Annealing (°C)
GAPDH	NM_017008.4	GAAGGTCGGTGTGAACGGATTTG	127	60
		CATGTAGACCATGTAGTTGAGGTCA		
MMP-1	NM_001134530.1	GCTTAGCCTTCCTTTGCTGTTGC	136	60
		GACGTCTTCACCCAAGTTGTAGTAG		
MMP-3	NM_133523	CTGGGCTATCCGAGGTCATG	77	60
		TGGACGGTTTCAGGGAGGC		
MMP-13	NM_133530	CAACCCTGTTTACCTACCCACTTAT	85	60
		CTATGTCTGCCTTAGCTCCTGTC		
COX-2	S67722	CGCATTCTTTGCCCAGCACTTCACT	190	60
		CACCTCTCCACCGATGACCTGATA		
iNOS	NM_012611.3	GCTCGGGCTGAAGTGGTATGC	127	60
		GAAGTCTCGGACTCCAATCTCGGT		
Bcl2	L14680	GGCTACGAGTGGGATACTGGAGAT	86	60
		CTCTCAGGCTGGAAGGAGAAGATG		
Bax	NM_017059	CCCCAGGACGCATCCACCAA	112	60
		GGGAGTCTGTATCCACATCAGCAA		

## Data Availability

The data used to support the findings of this study are included within the article.
